# A Rare Case of Progressive Palsy of the Lower Leg Caused by a Huge Lumbar Posterior Endplate Lesion after Recurrent Disc Herniation

**DOI:** 10.1155/2016/5963924

**Published:** 2016-08-28

**Authors:** Masatoshi Morimoto, Kosaku Higashino, Shinsuke Katoh, Tezuka Fumitake, Kazuta Yamashita, Fumio Hayashi, Yoichiro Takata, Toshinori Sakai, Akihiro Nagamachi, Koichi Sairyo

**Affiliations:** Department of Orthopedics, Institute of Health Biosciences, University of Tokushima Graduate School, Tokushima, Japan

## Abstract

A lesion of the lumbar posterior endplate is sometimes identified in the spinal canal of children and adolescents; it causes symptoms similar to those of a herniated disc. However, the pathology of the endplate lesion and the pathology of the herniated disc are different. We present a rare case of a 23-year-old woman who developed progressive palsy of the lower leg caused by huge lumbar posterior endplate lesion after recurrent disc herniation.

## 1. Introduction

A lesion of the lumbar posterior endplate is sometimes identified in the spinal canal of children and adolescents; it causes symptoms similar to those of a herniated disc [1–4]. However, the pathology of the endplate lesion and the pathology of the herniated disc are different [5]. The lesion of the posterior endplate lesion is usually not absorbed [6]; on the other hand lumbar disc herniation is usually absorbed. Our previous study described that the long-term outcome is good, in patients treated either conservatively or surgically for an endplate lesion [6]. We present a rare case of progressive palsy of the lower leg caused by huge lumbar posterior endplate lesion after recurrent disc herniation.

## 2. Case Report

**Figure 1:**
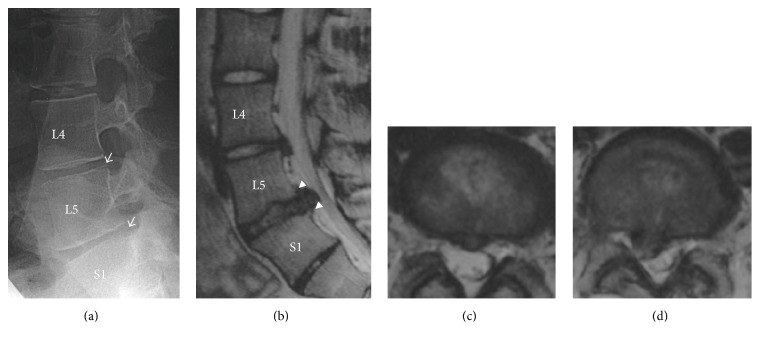
Radiographs showed lumbar posterior endplate lesions at L4/5 and L5/S1 at 14 years of age ((a), white arrows). Sagittal (b) and axial ((c) and (d)) T2-weighted MR images revealed L4/5 and L5/S1 lumbar disc herniation with endplate lesion. White arrow head showed irregular shape of the posterior end plate at the corner of S1 and L5 vertebrae.

This 14-year-old junior high school female had low back pain during ordinary daily activities. She was otherwise healthy without any other medical complications. She did not have hyperostosis disease, such as ossification of posterior longitudinal ligament or diffuse idiopathic skeletal hyperostosis. Neurological examination did not show obvious deficit at the first examination. Radiographic findings revealed lumbar disc degeneration with posterior end plate lesions at L4/5 and L5/S1 (Figure 1). She was treated with conservative therapy. The low back pain subsided after conservative therapy and she was able to return to ordinary daily life.

**Figure 2:**
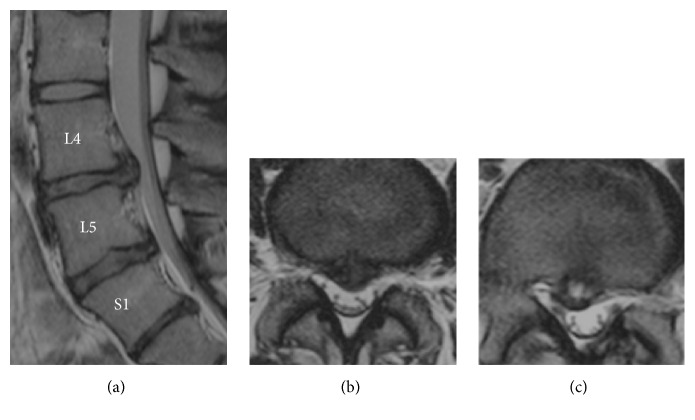
MRI showed posterior lumbar disc herniation connecting the nucleus pulposus tissue at L4/5 at the age of 18 years ((a) and (b)). The lumbar disc herniation at L5/S1 was absorbed, although the posterior endplate lesion still remained at L5/S1 ((a) and (c)).

At age 18 she had low back pain again. Radiographic findings showed a lumbar posterior lesion at L4/5 (Figure 2). We diagnosed lumbar disc herniation at L4/5. MRI showed posterior lumbar disc herniation connecting the nucleus pulposus tissue at L4/5. The lumbar disc herniation at L5/S1 was absorbed, although the posterior endplate lesion still remained at L5/S1. The endplate lesion at L5/S1 involved the cartilage or bony tissue of the growth plate and the tissue of the annulus and nucleus [5, 6]. She was treated with conservative therapy again. The low back pain disappeared completely. She worked at a metal plant factory after graduating from high school.

**Figure 3:**
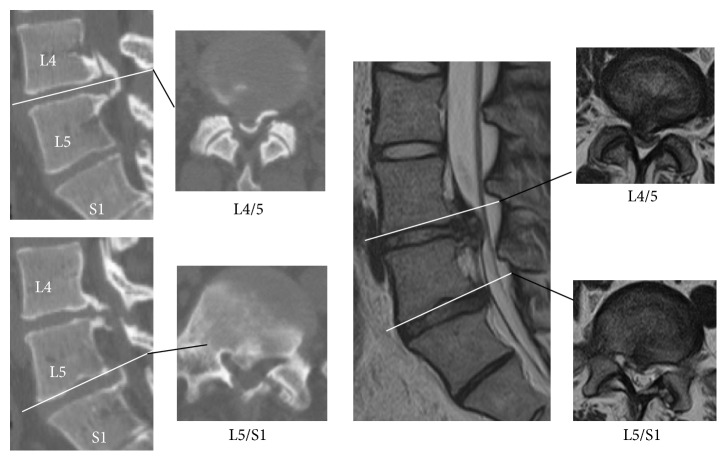
Computed tomography (CT) showed a huge ossification on the dorsal of annulus fibrosus of intervertebral disc at L4/5 at the age of 23 years. Ossification at L4/5 was larger than that at L5/S1. The ossification was consecutive with the posterior end plate at L4/5 and L5/S1. CT did not show an abnormal ossification of annulus fibrosus or posterior longitudinal ligament at the other levels. MRI revealed severe stenosis at L4/5 and the lesion of the posterior endplate lesion still remained at L5/S1.

At age 23 she visited her general physician due to muscle weakness of the right lower leg without low back pain. Manual muscle testing (MMT) of the right tibialis anterior muscle (TA) and that of the right extensor hallucis longus muscle (EHL) were both 4/5 at this first examination. On the seventh day her neurological deficit deteriorated. She had difficulty in walking with the right foot drop. On the thirteenth day she was referred to our hospital. At that time physical examination showed that MMT of both the right TA and the right EHL was 0/5. The straight leg raising test was restricted within 30 degrees. Sensory impairment at the right area of L4, L5, and S1 was presented. Computer tomography (CT) showed a huge ossification on the dorsal of annulus fibrosus of intervertebral disc at L4/5. CT did not show an abnormal ossification of annulus fibrosus or posterior longitudinal ligament at the other levels (Figure 3). MRI, compared to those taken when she was 18 years old, revealed severe stenosis at L4/5 and the lesion of the posterior endplate lesion still remained at L5/S1. She had progressive urinary retention.

She underwent surgery of L4 total laminectomy and L5 partial laminectomy with posterolateral fusion at L4/5. The huge ossification compressed the dura matter and adhered to L5 nerve root completely, so that the ossification was not able to be removed.

The postoperative course was satisfactory, her neurological deficit improved, and she had no difficulty in daily activities. Physical examination carried out 2 years later showed MMT of the TA to be 4/5 and EHL to be 2/5; sensory impairment had disappeared.

## 3. Discussion

The lumbar vertebral ring apophysis starts to ossify at about 6 years of age before ossification of ring apophysis. The vertebral ring apophysis appears as a secondary ossification center at approximately 10 years of age. Fusion to the vertebral body usually occurs between 17 and 20 years of age [5, 7–9]. A relative weak point exists between ring apophysis and the vertebral body until the fusion completes [10, 11]. Faizan et al. reported that the apophyseal ring experienced twice as much stress in the ossified stage as compared to the cartilaginous stage. This may be the cause of frequent fractures at the interface of bone and cartilage [12].

Terai et al. reported that the posterior region of the lumbar vertebrae in the pediatric model had higher stresses, as compared to the adult model. The most marked differences between the pediatric and adult models were found in the apophyseal ring [13].

In our previous study twenty-four consecutive patients with endplate lesions for 13.8 years in average were studied [5]. We concluded the long-term outcome for patients with a posterior endplate lesion is favorable. However radiographs showed degenerative changes at the lumbar disc and endplate lesion. Histological examination of the endplate lesion removed surgically showed an abnormal endplate with degeneration and some chondrocytes without a nucleus [6]. The endplate lesion seemed not able to remodel or not able to become resorbed.

The abnormal mechanical stress at the posterior lumbar lesion occurred twice at the age of 14 and 17 in this case. We think the huge ossification was a part of end plate lesion, because CT images of Figure 3 showed ossification was consecutive with the posterior end plate. Although we are not able to prove the pathological mechanism to induce the huge ossification of the end plate lesion, we speculated that multiple injuries might induce hyperossification of endplate lesion.

In summary, we report on a rare case of progressive palsy of the lower leg caused by huge lumbar posterior endplate lesion after recurrent disc herniation.
